# Research on trajectory tracking and body attitude control of autonomous ground vehicle based on differential steering

**DOI:** 10.1371/journal.pone.0273255

**Published:** 2023-02-08

**Authors:** Jie Tian, Mingfei Yang

**Affiliations:** College of Automobile and Traffic Engineering, Nanjing Forestry University, Jiangsu, Nanjing, China; Beijing Institute of Technology, CHINA

## Abstract

The differential steering can be used not only as the backup system of steer-by-wire, but also as the only steering system. Because the differential steering is realized through the differential moment between the coaxial left and right driving wheels, the sharp reduction of the load on the inner driving wheel will directly lead to the failure of the differential steering when the four-wheel independent drive electric vehicle approaches the rollover. Therefore, this paper not only realizes the trajectory tracking of autonomous ground vehicle through the differential steering, but also puts forward the body attitude control to improve the handling stability. Firstly, the dynamic and kinematic models of differential steering autonomous ground vehicle (DSAGV) and its roll model are established, and the linear three-degree of freedom vehicle model is selected as the reference model to generate the ideal body roll angle. Secondly, a model predictive controller (MPC) is designed to control the DSAGV to track the given reference trajectory, and obtain the required differential moment and the resulting front-wheel steering angle. Then, a sliding mode controller (SMC) is adopted to control the DSAGV to track the ideal body roll angle, and obtain the required roll moment. The simulation results show that the proposed MPC and SMC can not only make the DSAGV realize the trajectory tracking, but also achieve the body attitude control.

## Introduction

With the increasingly severe air pollution and energy crisis, electric vehicles (EVs) have attracted extensive attention from research institutions in various countries because of the characteristics of energy conservation and environmental protection [1], such as the energy management strategy [2], the fuel cell [3, 4], the battery SOC estimation [5–7], the different driving modes [8–11]. Relative speaking, due to the unstable operation of the engines, the strength requirements of parts are also higher [12, 13]. In addition, the emergence of four-wheel independent drive (4WID) EV with independent and controllable driving wheels [14–16] makes its dynamic control system more convenient and flexible [17–19]. At the same time, it also makes the differential steering to be possible through the coupling control of the left and right hub motors [20–22].

Based on the reference model, two different sliding mode controllers (SMCs) were proposed for the sliding steering and the differential steering, respectively. And the observer for the sideslip angle was also designed. The simulation results verified the effectiveness of the controllers, confirmed the feasibility of differential steering, and looked forward to the possibility of differential steering as the only steering system [23]. In order to realize the yaw control when the active front wheel steering fails completely, an integral sliding mode control strategy was proposed for the differential steering. Considering the unknown mismatched disturbances, a disturbance observer was designed. The nominal part of the controller was designed by the composite nonlinear feedback technique to suppress the overshoots and the steady-state errors when the tire force was saturated. An adaptive super-twisting control method was designed to solve the disturbances of the unknown boundaries to eliminate the chattering. The effectiveness of the designed control method was proved by CarSim-Simulink simulation results [24]. An H_∞_ output-feedback controller for the EV was used to realize the yaw control by the differential steering in case that the regular steering system failed. At the same time, the parameter uncertainties of the cornering stiffness and the external disturbances, as well as the uncertainty of the ideal steering angle and its difficulty to obtain were considered. And the front-wheel steering angle was realized by the differential moment of the front wheel hub motors. The CarSim-Simulink joint simulation results confirmed the effectiveness of the controller [25].

Literature [20] studied that when the conventional steering system failed, the lateral movement of the in-wheel motor-driven (IWMD) EV was realized by the differential steering, and the vertical loads of the wheels on both sides were balanced by considering the rollover characteristics of the vehicle, so as to ensure the efficiency of the differential steering. In order to solve the low cost measurement problem of the reference front-wheel steering angle and the lateral velocity, an H_∞_ observer-based controller was proposed to improve the vehicle handling stability. The effectiveness of the control strategy was evaluated by the joint simulation of CarSim and Simulink. With the consideration of the multiple unknown and mismatched disturbances of the steering system, an adaptive super-twisting control method was presented to realize the lane keeping for the four-wheel independently actuated autonomous vehicle through the differential steering. In order to improve the transient performance of the lane keeping control, a nonlinear sliding surface was designed to self-adaptively vary the system damping ratio. Based on a vision system, the estimations of the lane keeping errors and their time derivatives were made by a high-order sliding mode observer. The CarSim-Simulink simulation results proved the effectiveness and robustness of the controller to achieve lane keeping through the differential steering [21].

Literature [22] studied that after the sudden failure of the active front wheel steering, the path tracking control for an IWMD autonomous ground vehicle was realized through the differential steering. In this paper, an adaptive triple-step control method was proposed to realize the coordinated control of the transverse and longitudinal path tracking. The parameter uncertainties caused by the cornering stiffness and external disturbances were considered, and the effectiveness of the proposed control strategy was evaluated by the veDYNA-Simulink joint platform. In order to realize the trajectory tracking control of the autonomous ground vehicles when the steering motor failed completely, a model predictive controller (MPC) with the constraints of tire cornering angle and road adhesion was proposed to obtain the ideal front-wheel steering angle for the trajectory tracking of the traditional steering vehicle, and a nonsingular terminal sliding mode controller was designed to further realize the corresponding front-wheel steering angle with the differential moment. The joint simulation results of CarSim and Simulink confirmed the effectiveness of the trajectory tracking through the differential steering in the case of the complete failure of steering motor [26].

Normally, the vehicle will roll outward due to the action of the centrifugal force when turning. However, if the body can tilt to the turning direction like a bicycle or motor-cycle when making a turn, the torque generated by the vehicle gravity can reduce or even offset the torque generated by the centrifugal force, which can reduce the lateral load transfer ratio and the occupant perceived lateral acceleration, and prevent the failure of differential steering. In fact, as early as the 1980s, rail trains have begun to apply the active tilting technology [27], and the research showed that the inward roll angle of 1 ~ 2° can ensure that the rail train can pass through the curve at a high enough speed [28, 29]. For narrow commuter vehicle (NCV), which usually takes zero roll angle as the control target, the handling stability of the vehicle can be significantly improved as long as it tilts inward by 1 ~ 2° [30, 31].

To sum up, the feasibility of differential steering has been confirmed. At present, the academia has begun to consider the failure of differential steering caused by the sharp reduction of the load on the inner driving wheel when the vehicle approaches rollover, and try to use the differential steering to realize the trajectory tracking of autonomous ground vehicle. Based on the above research status, this paper takes the differential steering autonomous ground vehicle (DSAGV) as the research object, and makes the following innovations: (1) Referring to the active tilting technology of the rail trains and the NCVs, this paper puts forward the idea of body attitude control during the steering. That is, at the same time of realizing the differential steering control, it also realizes the active inward tilting control, so as to effectively avoid the failure of differential steering caused by the body outward tilting; (2) By analyzing the lateral stability during the steering, the ideal roll angle model of the vehicle body is established; (3) A MPC is adopted to control the differential steering to realize the trajectory tracking of the DSAGV, and the body inward tilting is control by the sliding mode controller (SMC). That is, the trajectory tracking and body attitude control of the DSAGV are achieved at the same time.

The structure of the article is as follows: Section 2 describes three vehicle models, including the model of the DSAGV, its roll model and the reference model. In Section 3, the trajectory tracking controller and the body attitude controller, i.e., the MPC and the SMC, are designed respectively. Section 4 gives the simulation results based on MATLAB/Simulink. And Section 5 is the conclusion.

## Vehicle model

Three different vehicle models, namely, the model of the DSAGV, its roll model and the reference model, are established in this section. Among them, the DSAGV model including the longitudinal, lateral and yaw dynamic models, and the kinematic model, will be controlled by the MPC to track the specific trajectory, and obtain the required differential moment and resulting front-wheel steering angle. Its roll model will be controlled by the SMC is used to track the ideal body roll angle, which is presented by the reference model. That is to say, the autonomous ground vehicle (AGV) model includes the model of the DSAGV and its roll model.

### Model of the DSAGV

**Fig 1:**
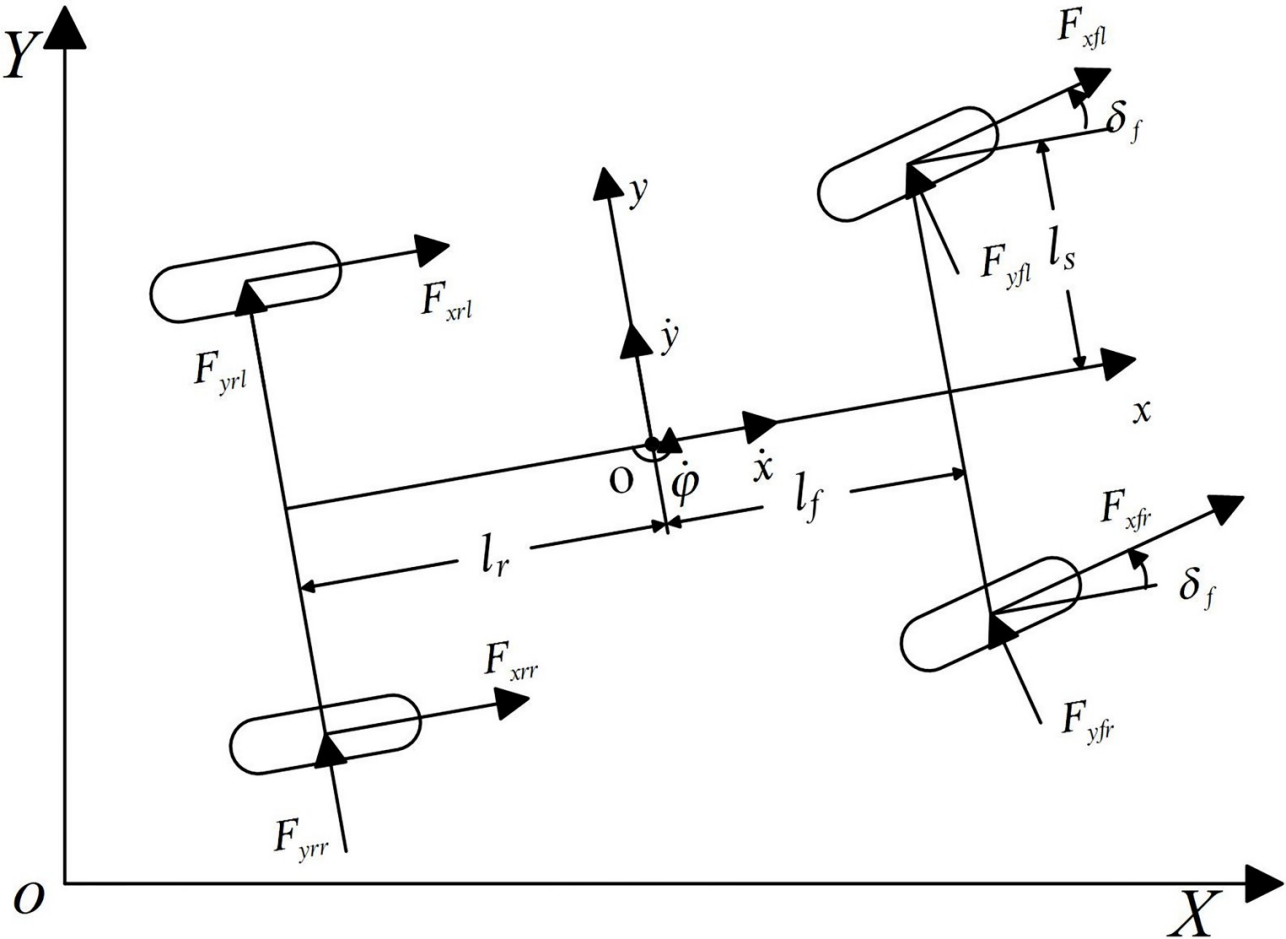
Planar dynamic model of the DSAGV.

Assuming that the DSAGV runs on a flat road and ignoring the load transfer, the vehicle planar model established is shown in Fig 1, where the *XOY* is the inertial coordinate system, and the *xoy* is the vehicle body coordinate system fixed at the vehicle centroid.

According to the Newton’s second law, the force balance equations along the x-axis, y-axis and around the z-axis are obtained as follows:

$$\left\{ \begin{array}{l} ma_{y}=m(\ddot{y}+\dot{x}\dot{\varphi })=F_{yfl}+F_{yfr}+F_{yrl}+F_{yrr}\\ m(\ddot{x}-\dot{y}\dot{\varphi })=F_{xfl}+F_{xfr}+F_{xrl}+F_{xrr}-(F_{yfl}+F_{yfr})\delta_{f}\\ I_{z}\ddot{\varphi }=l_{f}(F_{yfl}+F_{yfr})-l_{r}(F_{yrl}+F_{yrr})+\frac{l_{s}}{r}M_{z} \end{array}\right.,$$ (1)

$$F_{yfl}=F_{yfr}=k_{f}\alpha_{f},\;\alpha_{f}=\delta_{f}-\frac{\dot{y}+l_{f}\dot{\varphi }}{\dot{x}},\;F_{yrl}=F_{yrr}=k_{r}\alpha_{r},$$

$$\alpha_{r}=\frac{-\dot{y}+l_{r}\dot{\varphi }}{\dot{x}},\;F_{xfl}=F_{xfr}=k_{lf}s_{f},\;F_{xrl}=F_{xrr}=k_{lr}s_{r},$$

where *m* is the total mass of the vehicle, *a*_*y*_ is the inertial acceleration at the vehicle centroid, $\dot{y}$ and $\ddot{y}$ are the velocity and acceleration along the y-axis, $\dot{x}$ and $\ddot{x}$ are the velocity and acceleration along the x-axis, $\dot{\varphi }$ and $\ddot{\varphi }$ are the vehicle yaw angular velocity and angular acceleration, *M*_*z*_ is the differential driving torque of the left and right front wheels, *F*_*yfl*_, *F*_*yfr*_, *F*_*yrl*_ and *F*_*yrr*_ are respectively the lateral forces of the front, rear, left and right wheels, *F*_*xfl*_, *F*_*xfr*_, *F*_*xrl*_ and *F*_*xrr*_ are respectively the longitudinal forces of the front, rear, left and right wheels, *I*_*z*_ is the yaw rotary inertia of the vehicle, *l*_*f*_ and *l*_*r*_ are respectively the distances from the vehicle centroid to the front and rear axles, *l*_*s*_ is half of the wheel base, *k*_*f*_ and *k*_*r*_ are respectively the cornering stiffness of the front and rear wheels, *α*_*f*_ and *α*_*r*_ are respectively the sideslip angles of the front and rear wheels, *k*_*lf*_ and *k*_*lr*_ are respectively the longitudinal stiffness of the front and rear wheels, *s*_*f*_ and *s*_*r*_ are respectively the slip rates of the front and rear wheel.

The kinematic equation established from Fig 1 is

$$\left\{ \begin{array}{l} \dot{Y}=\dot{x}\sin\varphi +\dot{y}\cos\varphi \\ \dot{X}=\dot{x}\cos\varphi -\dot{y}\sin\varphi \end{array}.\right.$$ (2)

**Fig 2:**
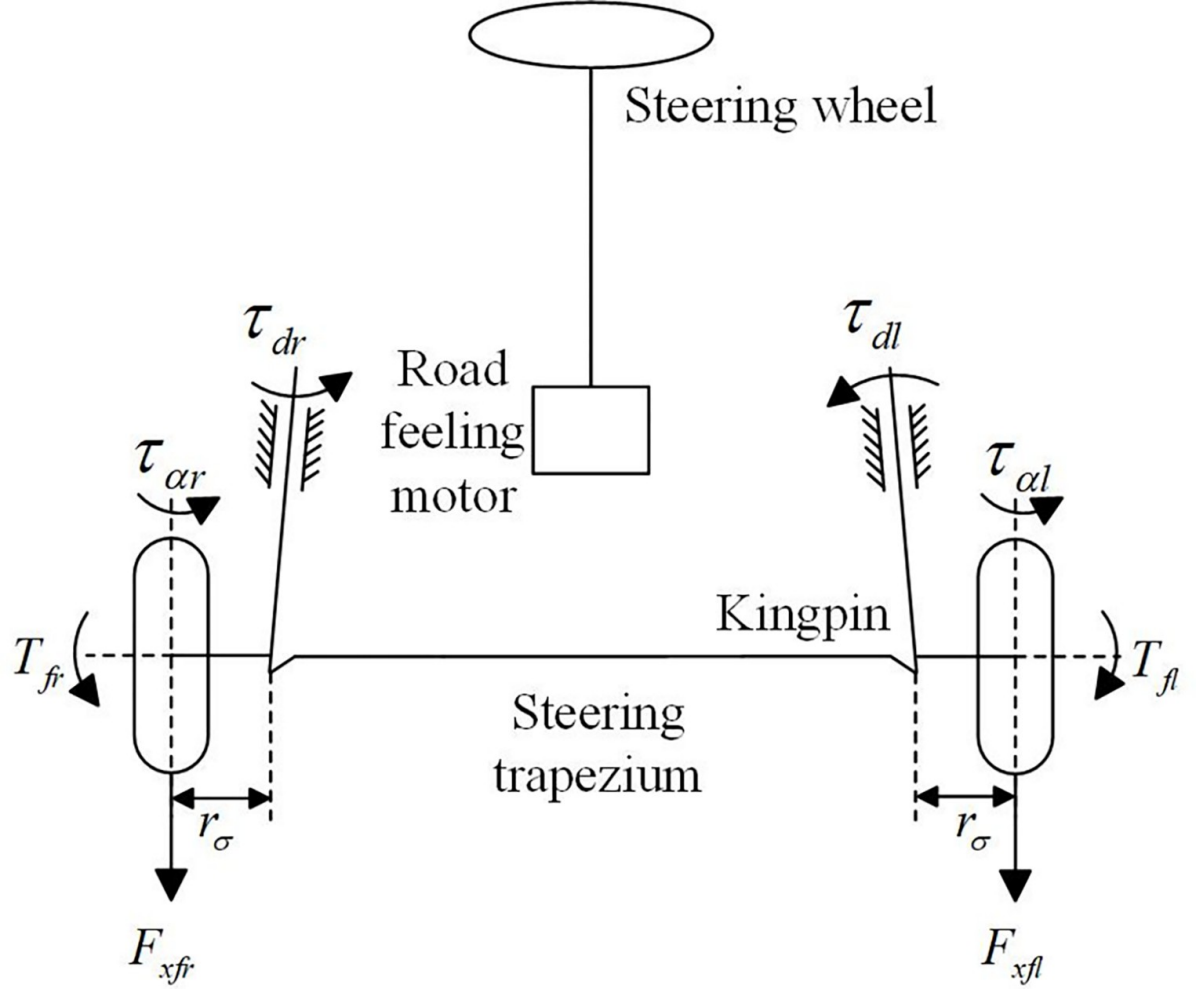
Differential steering system.

The four wheels of the distributed direct drive autonomous ground vehicle studied in this paper are equipped with hub motors, and the two front hub motors can realize the differential steering. The so-called differential steering refers to the steering through the difference of the driving torques between the coaxial left and right wheels. Its structure is shown in Fig 2. When the driver’s intention is provided to the electronic control unit (ECU) through the steering wheel, the ECU will give a command to the hub motors of the left and right front wheels respectively to generate two different driving forces. Due to the existence of kingpin offset, *r*_*σ*_, these two driving forces generate two torques around their respective kingpins, *τ*_*dr*_ and *τ*_*dl*_, to deflect the wheel to the longitudinal centerline of the vehicle. When the two torques are equal to each other, the vehicle goes straight. Otherwise, under the action of steering trapezoidal mechanism, the two wheels will deflect to the side of the wheel with the smaller torque, and realize the differential steering.

The dynamic equation of differential steering system can be expressed as

$$J_{e}{\ddot{\delta }}_{f}+b_{e}{\dot{\delta }}_{f}=\tau_{\alpha }+\frac{M_{z}}{r}r_{\sigma }-\tau_{f},$$ (3)

$$\tau_{\alpha }=\tau_{\alpha r}+\tau_{\alpha l}=2k_{f}\alpha_{f}l^{2}/3,\;M_{z}=T_{f}{}_{l}-T_{f}{}_{r}=(F_{xfl}-F_{xfr})r,$$

where *J*_*e*_ and *b*_*e*_ are respectively the equivalent moment of inertia and damping coefficient of the steering system, *τ*_*αl*_ and *τ*_*αr*_ are respectively the aligning torque of the left and right front wheels, *τ*_*a*_ is the total aligning torque of the front wheels, *T*_*fl*_ and *T*_*fr*_ are respectively the driving torque of the left and right front wheels, *F*_*xfl*_ and *F*_*xfr*_ are respectively the longitudinal force of the left and right front wheels, *r* is the tire rolling radius, *τ*_*f*_ is the friction force of the steering system, *l* is half of the contact path of the front wheel tire.

The mathematical model of the DSAGV obtained by synthesizing (1) ~ (3) is

$$\left\{ \begin{array}{l} {\dot{\delta }}_{f}=\frac{2l_{s}^{2}k_{f}}{3b_{e}}(\delta_{f}-\frac{\dot{y}+l_{f}\dot{\varphi }}{\dot{x}})+\frac{r_{\sigma }}{b_{e}r}M_{z}\\ \ddot{y}=-\dot{x}\dot{\varphi }+\frac{2}{m}[k_{f}(\delta_{f}-\frac{\dot{y}+l_{f}\dot{\varphi }}{\dot{x}})+k_{r}\frac{-\dot{y}+l_{r}\dot{\varphi }}{\dot{x}}]\\ \ddot{x}=\dot{y}\dot{\varphi }+\frac{2}{m}[k_{f}(\delta_{f}-\frac{\dot{y}+l_{f}\dot{\varphi }}{\dot{x}})\delta_{f}+k_{lf}s_{f}+k_{lr}s_{r}]\\ \ddot{\varphi }=\frac{1}{I_{z}}[2l_{f}k_{f}(\delta_{f}-\frac{\dot{y}+l_{f}\dot{\varphi }}{\dot{x}})-2l_{r}k_{r}\frac{-\dot{y}+l_{r}\dot{\varphi }}{\dot{x}}+\frac{l_{s}}{r}M_{z}]\\ \dot{Y}=\dot{x}\sin\varphi +\dot{y}\cos\varphi \\ \dot{X}=\dot{x}\cos\varphi -\dot{y}\sin\varphi \end{array}.\right.$$ (4)

Take the state variable as $\chi (t)={\left[\begin{array}{ccccccc} \delta_{f} & \dot{y} & \dot{x} & \varphi & \dot{\varphi } & Y & X \end{array}\right]}^{T}$, and input as *u*(*t*) = *M*_*z*_, Eq (4) can be expressed as

$$\dot{\chi }(t)=f(\chi (t),u(t)).$$ (5)

### Roll model of the DSAGV

**Fig 3:**
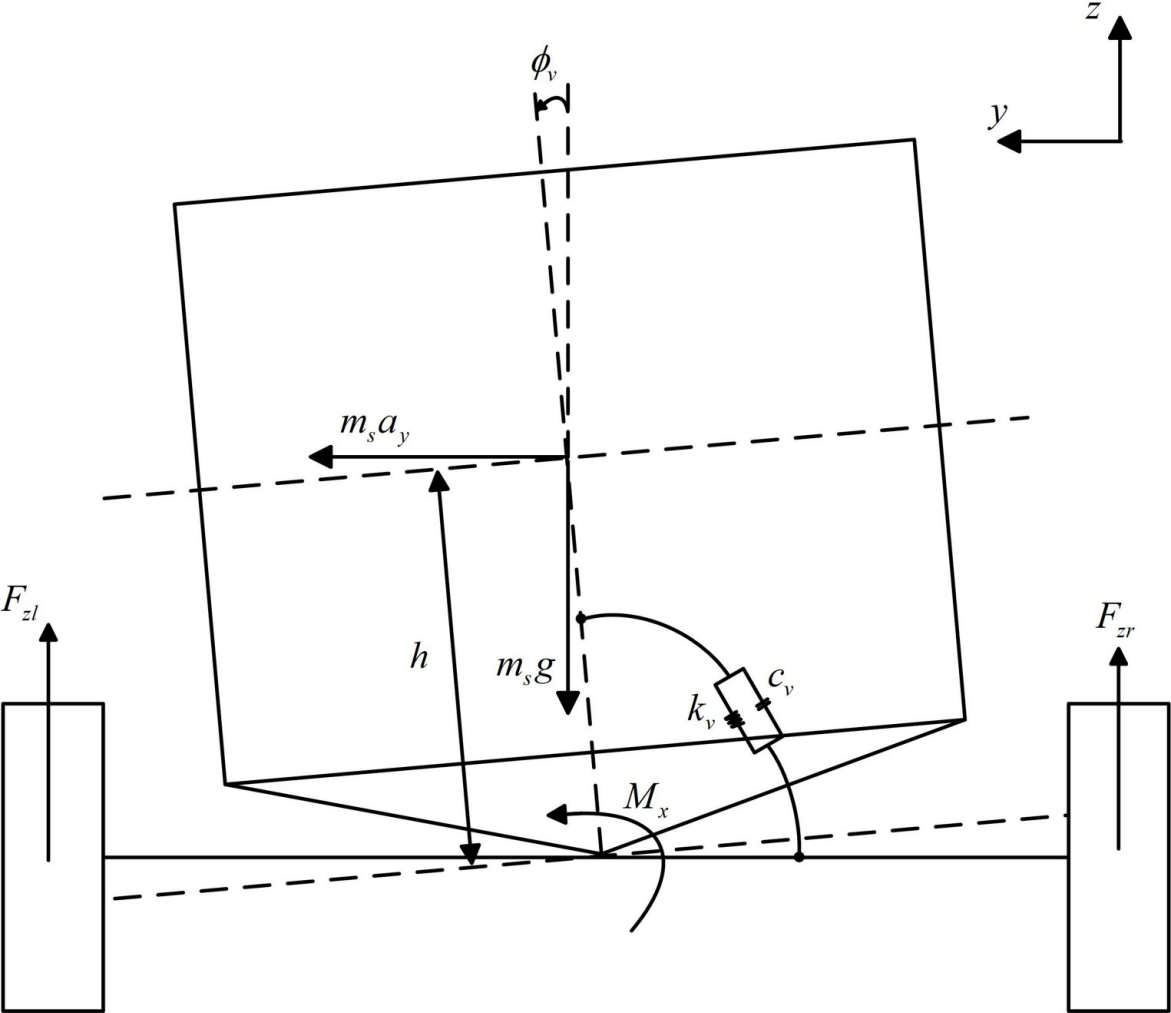
Roll dynamics model of the DSAGV (rear view).

Considering that when the vehicle is driving on a curve, the outward inclination of the body on the suspension will lead to a reduction in the vertical load of the inner wheel, especially when it occurs at the front wheel, it is very likely to directly lead to the failure of the differential steering, and even cause the rollover of the vehicle in serious cases. However, if the vehicle body can be actively tilted inward to the best angle through the active suspension to balance the gravity component and the centrifugal force, the effectiveness of the differential steering and the stability of the vehicle can be effectively guaranteed. Therefore, the established roll model of the DSAGV is shown in Fig 3.

The roll dynamic equation of the DSAGV established from Fig 3 is:

$$I_{x}{\ddot{\phi }}_{v}=m_{s}a_{y}h+m_{s}gh\phi_{v}-c_{v}{\dot{\phi }}_{v}-k_{v}\phi_{v}+M_{x},$$ (6)

where *I*_*x*_ is the roll rotary inertia of the vehicle, *ϕ*_*v*_, ${\dot{\phi }}_{v}$ and ${\ddot{\phi }}_{v}$ are respectively the roll angle, angular velocity and angular acceleration, *h* is the distance from the roll center height at the center of mass to the roll center height of the axle, *m*_*s*_ is the sprung mass of the vehicle, *g* is the gravity acceleration, *c*_*v*_ and *k*_*v*_ is the roll damping coefficient and roll stiffness coefficient of the suspension, and *M*_*x*_ is the roll moment provided by the active suspension.

By introducing the first equation in Eq (1), we can get

$${\ddot{\phi }}_{v}=\frac{1}{I_{x}}[\frac{2m_{s}k_{f}h}{m}(\delta_{f}-\frac{\dot{y}+l_{f}\dot{\varphi }}{\dot{x}})+\frac{2m_{s}k_{r}h}{m}(\frac{-\dot{y}+l_{r}\dot{\varphi }}{\dot{x}})+m_{s}gh\phi_{v}-c_{v}{\dot{\phi }}_{v}-k_{v}\phi_{v}+M_{x}].$$ (7)

### Reference model

The reference model selected in this paper is to provide the ideal body roll angle for the follow-up control strategy research, which requires that the model should not be too complex. Therefore, this paper adopts the linear three-degree of freedom (3-DOF) vehicle model as the reference model, that is, ignoring the influence of steering and suspension systems, only considering the lateral, yaw and roll motion of the vehicle, and linearizing the tire.

If the state space variable is $x_{d}(t)={\left[\begin{array}{cccc} \beta_{d} & \gamma_{d} & \phi_{v} & {\dot{\phi }}_{v} \end{array}\right]}^{T}$, and the system input is the front wheel angle, *δ*_*f*_, that is, *u*_2_(*t*) = *δ*_*f*_, the reference model can be expressed as:

$$\left\{ \begin{array}{l} {\dot{x}}_{d}=A_{d}x_{d}+B_{d}u_{2}\\ y_{d}=C_{d}x_{d}+D_{d}u_{2} \end{array},\right.$$ (8)

$$A_{d}=(\begin{array}{cccc} -\frac{2(k_{f}+k_{r})}{mv_{x}} & -1+\frac{2(-k_{f}l_{f}+k_{r}l_{r})}{mv_{x}^{2}} & 0 & 0\\ \frac{\left(-2k_{f}l_{f}+2k_{r}l_{r}\right)}{I_{z}} & -\frac{2k_{f}l_{f}^{2}+2k_{r}l_{r}^{2}}{I_{z}v_{x}} & 0 & 0\\ 0 & 0 & 0 & 1\\ \frac{2(k_{f}+k_{r})m_{s}h}{I_{x}m} & -\frac{2(-k_{f}l_{f}+k_{r}l_{r})m_{s}h}{I_{x}mv_{x}^{}} & \frac{m_{s}gh-k_{v}}{I_{x}} & -\frac{c_{v}}{I_{x}} \end{array}),$$

$$Bd=(\begin{array}{c} \frac{2k_{f}}{mv_{x}}\\ \frac{2k_{f}l_{f}}{I_{z}}\\ 0\\ -\frac{2k_{f}m_{s}h}{I_{x}m} \end{array}),\;Cd=[\begin{array}{cccc} 1 & 0 & 0 & 0\\ 0 & 1 & 0 & 0\\ 0 & 0 & 1 & 0\\ 0 & 0 & 0 & 1 \end{array}],\;Dd=[\begin{array}{c} 0\\ 0\\ 0\\ 0 \end{array}],$$

where *β*_*d*_ and *γ*_*d*_ are the ideal sideslip angle and the yaw rate of the center of mass.

Referring to the body tilting control of rail trains and the NCVs, this paper proposes to make the vehicle body actively tilt inward when the vehicle turns. In other words, it is necessary to make the torque generated by the gravity on the vehicle body balance with the torque generated by the steering centrifugal force, so as to avoid the rollover of the vehicle and the failure of the differential steering. In addition, it can also make the lateral acceleration perceived by passengers equal or close to zero.

For the reference model, the torque generated by the steering centrifugal force is

$$M_{S}=m_{s}v_{x}({\dot{\beta }}_{d}+\gamma_{d})h\cos\phi_{vd}.$$ (9)

The roll moment due to the gravity is

$$M_{G}=m_{s}gh\sin\phi_{v}.$$ (10)

From *M*_*S*_ = *M*_*G*_, the ideal body roll angle can be obtained as

$$\phi_{vd}=\arctan\frac{v_{x}({\dot{\beta }}_{d}+\gamma_{d})}{g}.$$ (11)

That is, we can obtain the ideal body roll angle according to Eq (8) and Eq (11) in this section.

Here, the occupant perceived lateral acceleration and the lateral load transfer ratio are employed to evaluate the effect of active vehicle body inward tilting. Among them, the lateral acceleration perceived by passengers has a certain impact on the ride comfort of the vehicle, which is mainly composed of the lateral acceleration, the component of gravity acceleration and the vehicle body roll acceleration, and can be expressed as:

$$a_{per}=\ddot{y}\cos\phi_{v}+h{\dot{\phi }}_{v}-g\sin\phi_{v}.$$ (12)

The Lateral load transfer ratio (*LTR*) is a commonly used index for predicting vehicle non-tripping rollover [18], which can be expressed as

$$LTR=\frac{m_{s}\ddot{y}h+I_{x}{\ddot{\phi }}_{v}-m_{s}gh\phi_{v}}{m_{s}gl_{s}}.$$ (13)

## Trajectory tracking and attitude control

### Problem statement

**Fig 4:**
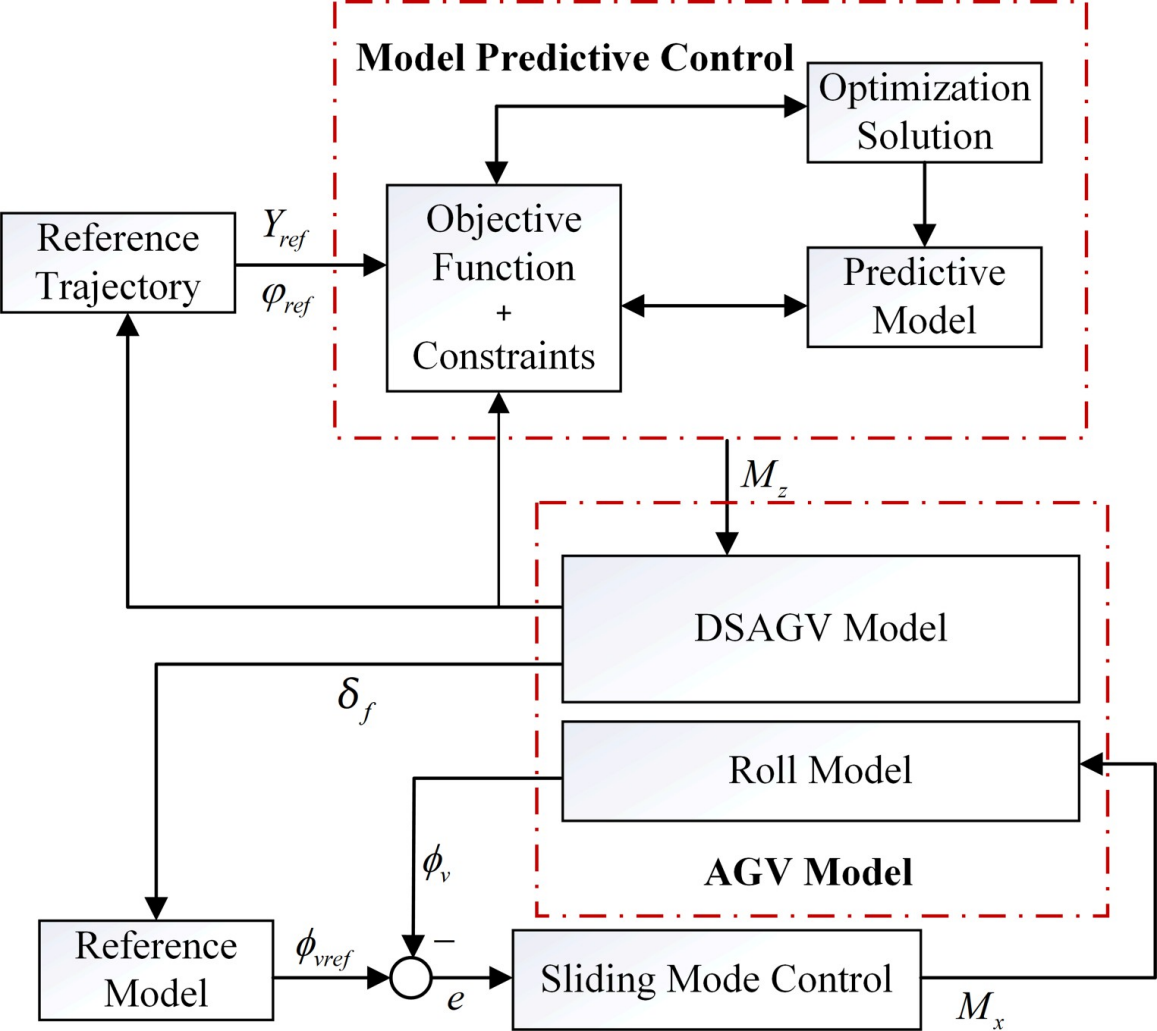
Control block diagram.

The control block diagram shown in Fig 4 is proposed in this paper. In order to make the DSAGV track the specific driving trajectory, a MPC is designed to realize the differential steering and obtain the required differential moment, *M*_*z*_. Specifically, the relevant parameters derived from the DSAGV model shown in Eq (5) are provided to obtain the reference trajectory, *Y*_*ref*_ and *φ*_*ref*_, required by the objective function, and the relevant state variables, *χ*, are provided to the constraints at the same time, then the differential moment, *M*_*z*_, required by the DSAGV to track a specific trajectory can be obtained through optimization. In addition, the SMC is adopted to make the roll model of the DSAGV shown in Eq (7) have the same body roll angle as the reference model, and obtain the roll moment, *M*_*x*_. By inputting the obtained differential and roll moments, *M*_*z*_ and *M*_*x*_, to the DSAGV model and its roll model respectively, the state variables, *χ*, the front wheel steering angle, *δ*_*f*_ and the body roll angle, *ϕ*_*v*_ can all be obtained. Among them, the front wheel steering angle, *δ*_*f*_, is provided to the reference model shown in Eq (8) to obtain the ideal body roll angle, *ϕ*_*vref*_. However, *ϕ*_*v*_ and *ϕ*_*vref*_ are used to generate the tracking error required by the SMC. In this way, a closed-loop control system can be formed.

### Design of the MPC

The MPC designed in this section is used to control the DSAGV to track a specific driving trajectory and obtain the required differential moment, *M*_*z*_. Since the reference trajectory cannot give the information of all state points, the linear equation of Eq (5) is obtained by the following method.


$$\left\{ \begin{array}{l} \dot{\chi }(t)=A(t)\chi (t)+B(t)u(t)\\ y(t)=C(t)\chi (t) \end{array},\right.$$ (14)

$$A(t)=\frac{\partial f}{\partial \chi }=[\begin{array}{ccccccccc} \frac{\partial f_{1}}{\partial \delta } & \frac{\partial f_{1}}{\partial \dot{y}} & \frac{\partial f_{1}}{\partial \dot{x}} & \frac{\partial f_{1}}{\partial \varphi } & \frac{\partial f_{1}}{\partial \dot{\varphi }} & \frac{\partial f_{1}}{\partial \phi_{v}} & \frac{\partial f_{1}}{\partial {\dot{\phi }}_{v}} & \frac{\partial f_{1}}{\partial Y} & \frac{\partial f_{1}}{\partial X}\\ \vdots & \vdots & \vdots & \vdots & \vdots & \vdots & \vdots & \vdots & \vdots \\ \frac{\partial f_{7}}{\partial \delta } & \frac{\partial f_{7}}{\partial \dot{y}} & \frac{\partial f_{7}}{\partial \dot{x}} & \frac{\partial f_{7}}{\partial \varphi } & \frac{\partial f_{7}}{\partial \dot{\varphi }} & \frac{\partial f_{7}}{\partial \phi_{v}} & \frac{\partial f_{7}}{\partial {\dot{\phi }}_{v}} & \frac{\partial f_{7}}{\partial Y} & \frac{\partial f_{7}}{\partial X} \end{array}],B(t)=\frac{\partial f}{\partial u}=[\begin{array}{cc} \frac{\partial f_{1}}{\partial M_{z}} & \frac{\partial f_{1}}{\partial M_{x}}\\ \vdots & \vdots \\ \frac{\partial f_{7}}{\partial M_{z}} & \frac{\partial f_{7}}{\partial M_{x}} \end{array}],$$

$$C(t)=[\begin{array}{ccccccc} 0 & 0 & 0 & 1 & 0 & 0 & 0\\ 0 & 0 & 0 & 0 & 0 & 1 & 0 \end{array}].$$

The first-order difference quotient method is adopted to discretize Eq (14), and the following discrete state space expression can be obtained as

$$\left\{ \begin{array}{l} \xi (k+1|t)=\tilde{A}(k)\xi (k|t)+\tilde{B}(k)\Delta u(k|t)\\ \eta (k|t)=\tilde{C}(k)\xi (k|t) \end{array},\right.$$ (15)

$$\tilde{A}(k)=[\begin{array}{cc} A(k) & B(k)\\ 0_{1\times 7} & I_{1} \end{array}],\;\tilde{B}(k)=[\begin{array}{c} B(k)\\ I_{1} \end{array}],\;\tilde{C}(k)=[\begin{array}{ccccccc} 0 & 0 & 0 & 1 & 0 & 0 & 0\\ 0 & 0 & 0 & 0 & 0 & 1 & 0 \end{array}].$$

The predictive output equation of the system can be derived from (15)

Y(k+1|t)=ψξ(k|t)+ΘΔU(k|t), (16)

$$Y(k+1|t)=[\begin{array}{c} \eta (k+1|t)\\ \eta (k+2|t)\\ \vdots \\ \eta (k+N_{p}|t) \end{array}],\;\Delta U(k|t)=[\begin{array}{c} \Delta u(k|t)\\ \Delta u(k+1|t)\\ \vdots \\ \Delta u(k+N_{c}|t) \end{array}],\;\psi =[\begin{array}{c} C{\tilde{A}}_{k,1}\\ C{\tilde{A}}_{k,2}\\ \vdots \\ C{\tilde{A}}_{k,N_{c}}\\ \vdots \\ C{\tilde{A}}_{k,N_{p}} \end{array}],\;\Theta =[\begin{array}{cccc} C{\tilde{B}}_{k} & 0 & \cdots & 0\\ C{\tilde{A}}_{k}{\tilde{B}}_{k} & C{\tilde{B}}_{k} & \cdots & 0\\ \vdots & \vdots & \ddots & \vdots \\ C{\tilde{A}}_{k,N_{c}}{\tilde{B}}_{k} & C{\tilde{A}}_{k,N_{c}-1}{\tilde{B}}_{k} & \cdots & C{\tilde{B}}_{k}\\ \vdots & \vdots & \ddots & \vdots \\ C{\tilde{A}}_{k,N_{p}-1}{\tilde{B}}_{k} & C{\tilde{A}}_{k,N_{p}-2}{\tilde{B}}_{k} & \cdots & C{\tilde{A}}_{k,N_{p}-N_{c}-1}{\tilde{B}}_{k} \end{array}],$$

where *N*_*p*_ is the prediction time domain, *N*_*c*_ is the control time domain.

In order to ensure that the autonomous ground vehicle can track the desired trajectory quickly and smoothly, the objective function shown below is designed.

$$J(k)={\sum_{i=1}^{N_{p}}\left\|\Delta \eta (k+i|t)\right\|}_{Q}^{2}+{\sum_{i=1}^{N_{c}-1}\left\|\Delta u(k+i|t)\right\|}_{R}^{2}+\rho \varepsilon^{2},$$ (17)

where Δ*η*(*k*+*i*|*t*) is the tracking error between the state of the actual system and that of the reference system, Δ*u*(*k*+*i*|*t*) is the control increment of the differential moment, *Q*, *R*, and *ρ* are the weight coefficients, *ε* is the relaxation factor.

Eq (17) can also be transformed into the following standard quadratic form.

$$J(\xi (t),u(t-1),\Delta U(t))={\left[\Delta U{\left(t\right)}^{T},\varepsilon \right]}^{T}H[\Delta U{\left(t\right)}^{T},\varepsilon ]+G[\Delta U{\left(t\right)}^{T},\varepsilon ],$$ (18)

$$H=[\begin{array}{cc} \Theta^{T}Q\Theta +R & 0\\ 0 & \rho \end{array}],G=[\begin{array}{cc} 2e_{1}^{T}Q\Theta & \end{array}0],$$

where *e*_1_ is the tracking error in the prediction time domain.

The constraints are designed as

$$\left\{ \begin{array}{l} \Delta U_{\min}\leq \Delta U\leq \Delta U_{\max}\\ U_{\min}\leq A\Delta U+U\leq U_{\max}\\ y_{h,\min}\leq y_{h}\leq y_{h,\max}\\ y_{s,\min}-\varepsilon \leq y_{s}\leq y_{s,\max}+\varepsilon \end{array}.\right.$$ (19)

where Δ*U*_min_ and Δ*U*_max_ are the lower and upper boundary values of control input increment, *U*_min_ and *U*_max_ are the lower and upper boundary values of control input, *A* is the Kronecker product of an unit lower triangular matrix and an unit matrix, *y*_*h*,min_ and *y*_*h*,max_ are the lower and upper boundary values of the hard constraint of output, *y*_*s*,min_ *y*_*s*,max_ are the lower and upper boundary values of the soft constraint of output.

Taking Eq (18) as the objective function and Eq (19) as the constraint condition, the quadratic programming problem can be solved through the quadprog function of MATLAB, and the differential moment required by the DSAGV, *M*_*z*_, to track the reference trajectory can be obtained.

### Design of the SMC

Sliding mode control is a control method with strong robustness, which has the advantages of fast response and insensitive to external changes and disturbances. The chattering of system can be effectively reduced through the control of exponential reaching law. The vehicle body inward tilting controller based on the sliding mode control theory is designed in this section.

According to the roll model of the DSAGV and the reference model, the tracking error of the roll angle is assumed to be *e*, which can be expressed as

$$e=\phi_{vref}-\phi_{v}$$ (20)

The following switching function is selected for the control of the body roll angle.

$$s=ce+\dot{e},$$ (21)

where *c* is the controller parameter that needs to meet the Hurwitz condition, and its value is greater than zero.

Take $\dot{s}=0$, then

$$\dot{s}=-\frac{1}{I_{x}}[\frac{2m_{s}k_{f}h}{m}(\delta_{f}-\frac{\dot{y}+l_{f}\dot{\varphi }}{\dot{x}})+\frac{2m_{s}k_{r}h}{m}(\frac{-\dot{y}+l_{r}\dot{\varphi }}{\dot{x}})+(m_{s}gh-k_{v})\phi_{v}-c_{v}{\dot{\phi }}_{v}+M_{x}]=0$$ (22)

The equivalent control of the vehicle body inward tilting can be designed as follows:

$$M_{xeq}=-\frac{2m_{s}k_{f}h}{m}(\delta_{f}-\frac{\dot{y}+l_{f}\dot{\varphi }}{\dot{x}})-\frac{2m_{s}k_{r}h}{m}(\frac{-\dot{y}+l_{r}\dot{\varphi }}{\dot{x}})-(m_{s}gh-k_{v})\phi_{v}+c_{v}{\dot{\phi }}_{v}+I_{x}(c\dot{e}+{\ddot{\phi }}_{vref})$$ (23)

In order to ensure that the reaching condition of the sliding mode is established, that is, $s\dot{s}\leq -\eta |s|$ and *η*>0, the switching control of the vehicle body inward tilting is designed as follows:

$$M_{xsw}=I_{x}\eta \mathrm{sgn}(s).$$ (24)

Then the sliding mode control law of the vehicle body inward tilting is composed of equivalent control term and switching control term, that is

$$M_{x}=M_{xeq}+M_{xsw}.$$ (25)

Substitute Eq (25) into Eq (22) to obtain

$$\dot{s}=c\dot{e}+{\ddot{\phi }}_{vref}-\frac{1}{I_{x}}[\begin{array}{l} \frac{2m_{s}k_{f}h}{m}(\delta_{f}-\frac{\dot{y}+l_{f}\dot{\varphi }}{\dot{x}})+\frac{2m_{s}k_{r}h}{m}(\frac{-\dot{y}+l_{r}\dot{\varphi }}{\dot{x}})+(m_{s}gh-k_{v})\phi_{v}-c_{v}{\dot{\phi }}_{v}+\\ -\frac{2m_{s}k_{f}h}{m}(\delta_{f}-\frac{\dot{y}+l_{f}\dot{\varphi }}{\dot{x}})-\frac{2m_{s}k_{r}h}{m}(\frac{-\dot{y}+l_{r}\dot{\varphi }}{\dot{x}})-(m_{s}gh-k_{v})\phi_{v}+c_{v}{\dot{\phi }}_{v}\\ +I_{x}(c\dot{e}+{\ddot{\phi }}_{vref})+I_{x}\eta \mathrm{sgn}(s) \end{array}]=-\eta \mathrm{sgn}(s)$$ (26)

Then,

$$s\dot{s}=-s\eta \mathrm{sgn}(s)=-\eta |s|\leq 0$$ (27)

## Simulation results and analysis

In order to verify the effectiveness of the designed MPC and SMC, the trajectory tracking and attitude control of the DSAGV are simulated in Matlab/Simulink environment. The vehicle parameters used in the simulation are: *m* = 1843*kg*, *m*_*s*_ = 1723*kg*, *h* = 0.54*m*, *g* = 10 *m*/*s*^2^, *b*_*e*_ = 100 *N*, *l* = 0.0368 *m*, *r*_*σ*_ = 0.0754*m*, *R* = 0.298*m*, *I*_*z*_ = 4175 *kg*∙*m*^2^, *I*_*x*_ = 1243.1 *kg*∙*m*^2^, *l*_*f*_ = 1.232*m*, *l*_*r*_ = 1.468*m*, *l*_*s*_ = 0.74*m*, *k*_*v*_ = 48000 *N*/*m*, *c*_*v*_ = 6000 *Ns*/*m*∙*rad*.

### Double lane change maneuver

In this paper, the double lane trajectory which consists of a reference lateral position, *Y*_*ref*_, and a reference yaw angle, *φ*_*ref*_, is used as the tracking trajectory of the differential steering autonomous ground vehicle. And the specific expression is as follows:

$$\left\{ \begin{array}{l} Y_{ref}(X)=\frac{d_{y1}}{2}(1+\tanh(z_{1}))-\frac{d_{y2}}{2}(1+\tan(z_{2}))\\ \varphi_{ref}(X)=\arctan(d_{y1}{\left(\frac{1}{\cosh(z_{1})}\right)}^{2}(\frac{1.2}{d_{x1}})-d_{y2}{\left(\frac{1}{\cosh(z_{2})}\right)}^{2}(\frac{1.2}{d_{x2}})) \end{array},\right.$$ (28)

where $z_{1}=\frac{2.4}{25}(X-27.19)-1.2$, $z_{2}=\frac{2.4}{21.95}(X-56.45)-1.2$, *d*_*x*1_ = 25, *d*_*x*2_ = 21.95, *d*_*y*1_ = 4.05, *d*_*y*2_ = 4.05.

**Fig 5:**
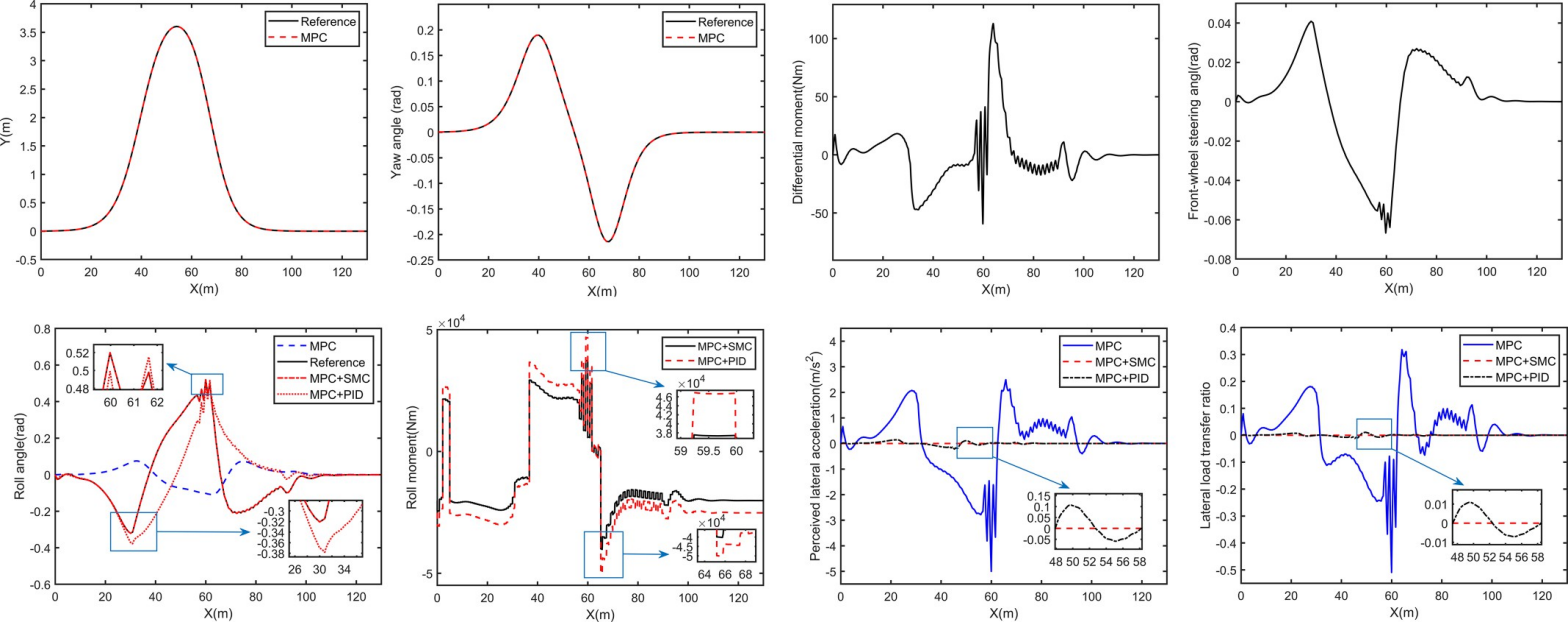
Curves of double lane change simulation. (A) Trajectory; (B) Yaw angle; (C) Differential moment; (D) Front-wheel steering angle; (E) Body roll angle; (F) Roll moment; (G) Occupant perceived lateral acceleration; (H) Lateral load transfer ratio.

Set the vehicle speed as 60 km/h, the curves of the simulation results are shown in Fig 5.

The trajectory curve of the DSAGV with the MPC is obtained by the simulation, as shown in Fig 5A and 5B. It can be seen from Fig 5A and 5B that the DSAGV can track the lateral position and yaw angle of the reference trajectory very well, which show that the MPC designed in this paper for trajectory tracking is effective.

The differential moment required by the DSAGV to track the reference trajectory and the resulting front-wheel steering angle are shown in Fig 5C and 5D, respectively. It can be seen that both of them change with the X position of the vehicle.

It can be seen from Fig 5C and 5D that the differential moment slowly increases to the first peak value of 18.12Nm after a small fluctuation of one cycle from zero, then decreases to zero when X = 30.17m. Since the differential moment is positive during this period, the corresponding front-wheel steering angle increases slowly after a small fluctuation of one cycle from zero, and reaches the peak value of 0.04072rad when X = 30.17m. After that, the differential moment continues to decrease, increases again after reaching the first minimum value of -47.02Nm, rapidly increases to the second peak value of 111.8Nm after shaking around 0 for 3 cycles, and decreases to be zero again when X = 71.25m. Therefore, the corresponding front-wheel steering angle firstly decreases all the time. After three cycles of small vibration, it reaches the first minimum value of -0.0667rad at X = 59.77m, increases rapidly, and reaches the second peak value of 0.02593rad at X = 71.25m. After that, the differential moment becomes negative and finally becomes zero after several oscillations, and the front-wheel steering angle decreases all the time and finally becomes zero.

Fig 5E shows the ideal body roll angle and the body roll angles generated by the DSAGV with and without the roll control. It can be seen that the body roll angle generated by the DSAGV controlled only by the MPC increases from 0 with the change of X. After reaching the first peak value of 0.079rad at X = 34.16, it decreases to the minimum value of -0.127rad, and then increases again to the second peak value of 0.069rad and gradually decreases to zero. While with the change of X, the ideal body roll angle decreases from zero to the first minimum value of -0.403rad after a small fluctuation of one cycle, increases again to the peak value of 0.6011rad, decreases again to the second minimum value of -0.27rad, and then gradually increases to zero.

In other words, the direction of the ideal body roll angle is almost opposite to that of the DSAGV controlled by the MPC only, and the amplitude of the former is significantly larger than that of the latter. In addition, it also can be seen that the body roll angle produced by the DSAGV jointly controlled by the MPC and the SMC is almost exactly consistent with the ideal one. Therefore, the conclusion can be drawn that in order to reduce or offset the torque generated by gravity using the torque generated by centrifugal force, the SMC does make the vehicle body actively tilt inward when turning.

The roll moment required by the DSAGV to track the ideal body roll angle is shown in Fig 5F. Its trend is generally consistent with that of the ideal body roll angle, and the maximum and the minimum values are 38920Nm and -43640Nm, respectively. The resulting occupant perceived lateral acceleration and lateral load transfer ratio are shown in Fig 5G and 5H, respectively. It can be seen that both of them generated by the DSAGV controlled by the MPC only change with the change of X and their maximum absolute value reaches to 4.46 m/s^2^ and 0.376, while those of the DSAGV controlled by the MPC and the SMC is almost zero. It can be seen that the roll control proposed in this paper based on the differential steering controller is feasible, which can not only make the DSAGV have an ideal body roll angle, but also reduce the occupant perceived lateral acceleration and lateral load transfer ratio to zero.

To sum up, compared with MPC control, the MPC and the SMC can realize the trajectory tracking and body attitude control of the DSAGV.

### 90° turning maneuver

In order to verify the effect of the controllers at low speed, a quarter circle trajectory with a turning radius of 100m is selected as the tracking trajectory of the DSAGV under 90° turning condition.

The specific expression of the trajectory is as follows:

$$\left\{ \begin{array}{l} \varphi_{ref}=\frac{v\tan\delta_{d}}{l}t\\ X_{ref}=100\sin\varphi \\ Y_{ref}=100-100\cos\varphi \end{array},\right.$$ (29)

where *δ*_*d*_ is the desired front-wheel steering angle.

**Fig 6:**
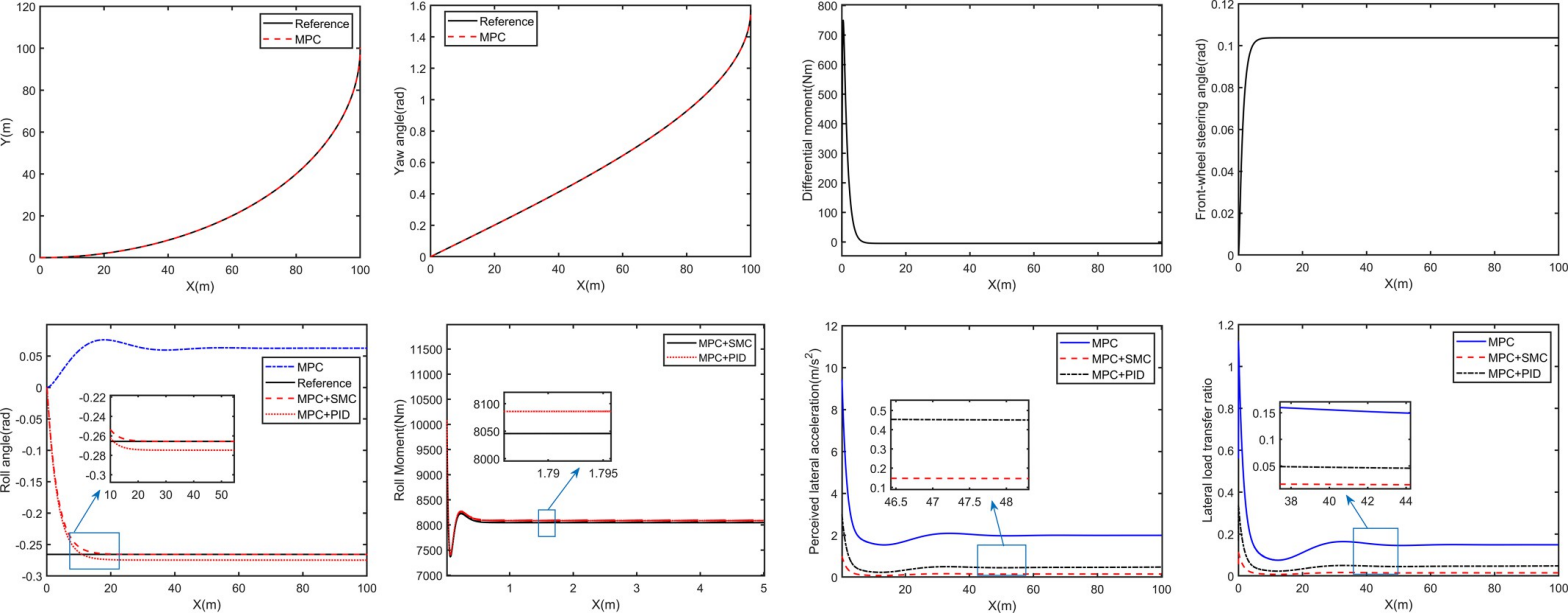
**90**^**o**^ **turning simulation.** (A) Trajectory; (B) Yaw angle; (C) Differential moment; (D) Front-wheel steering angle; (E) Body roll angle; (F) Roll moment; (G) Occupant perceived lateral acceleration; (H) Lateral load transfer ratio.

Select the vehicle speed and *δ*_*d*_ as 30 km/h and 0.1036rad, the curves of the simulation results are shown in Fig 6.

The trajectory of the DSAGV with the MPC is shown in Fig 6A and 6B. It can be seen from Fig 6B that the controlled DSAGV can well track the lateral position and yaw angle of the reference trajectory.

The differential moment required to track the reference trajectory and the resulting front-wheel steering angle are shown in Fig 6C and 6D, respectively. It can be seen that the differential moment increases rapidly from zero to the peak value of 742.8Nm and then decreases rapidly. When X = 6.046m, the differential moment becomes zero. The resulting front-wheel steering angle increases rapidly to the peak value of 0.1036 rad, and then remains unchanged.

Fig 6E shows the comparison curve of ideal body roll angle and body roll angle generated by the DSAGV with and without roll control. It can be seen that the extreme value of the ideal body inclination is -0.26rad, and that generated by the DSAGV controlled by the MPC is 0.065rad. The two directions are opposite, and the amplitude of the former is obviously larger than that of the latter. It can be seen that in order to reduce the torque generated by gravity or offset the torque generated by centrifugal force, it is necessary to make the vehicle actively tilt inward at a relatively large angle when turning. In addition, it also can be that after X = 20m, the body roll angle produced by the DSAGV jointly controlled by the MPC and the SMC is almost completely consistent with the ideal body roll angle. The roll moment required by the DSAGV to track the ideal body inclination is shown in Fig 6F. As can be seen from the figure, the roll moment rapidly increased to the peak value of 10650Nm, and then stabilized at 8010Nm after a small fluctuation.

The resulting occupant perceived lateral acceleration and lateral load transfer rate are shown in Fig 6G and 6H, respectively. It can be seen that both of them generated by the DSAGV controlled by the MPC increase rapidly to the peak value at 0 seconds, and the peak values are 10.45 m/s^2^ and 1.108, respectively. After that, both of them decreased rapidly and finally stabilized at 1.996 m/s^2^ and 0.16 respectively after a small fluctuation. Those of the DSAGV jointly controlled by the MPC and the SMC are 0.1632 m/s^2^ and 0.01462 respectively. It can be seen that the roll controller proposed in this paper based on the differential steering controller is feasible.

To sum up, compared with MPC control, the MPC and the SMC can realize the trajectory tracking and body attitude control of the DSAGV.

## Conclusion

For the DSAGV, the trajectory tracking control and the body attitude control are carried out at the same time.

1. The DSAGV model is established, the model prediction equation is deduced, the objective function and constraints are constructed. The MPC is used to control the trajectory tracking of the DSAGV, and the required differential moment and the resulting front-wheel steering angle are obtained. And the ideal body roll angle is achieved by the reference model according to the front-wheel steering angle.
2. The roll model of DSAGV is established and the ideal body roll angle is studied. Based on the sliding mode control theory, the vehicle body inclination controller is designed to realize the attitude tracking of the ideal body roll angle for the DSAGV, and the required roll moment is obtained.
3. The effectiveness of the differential steering controller and the body inclination controller proposed in this paper, that is the MPC and the SMC, is verified by the simulation of two working conditions.
